# Effects of mating on reproductive performance of *Coccophagus japonicus* Compere (Hymenoptera: Aphelinidae)

**DOI:** 10.1038/s41598-021-85351-x

**Published:** 2021-03-15

**Authors:** Xian Li, Shunzhang Shen, Yueguan Fu, Junyu Chen, Lei Li, Dongyin Han, Junhong Zhu, Fangping Zhang

**Affiliations:** 1 Environment and Plant Protection Institute, Chinese Academy of Tropical Agricultural Sciences, Haikou, 571101 China; 2 College of Plant Protection, Hainan University, Haikou, 570228 China

**Keywords:** Plant sciences, Zoology

## Abstract

*Coccophagus japonicus* Compere, an endoparasitoid of *Parasaissetia nigra* Nietner, has great potential for biological control. To assess the influence of mating on the reproductive performance of this parasitoid, we examined the effects of mating on ovarian development, female longevity and number of eggs laid. The results showed that the egg volume in the ovary of *C. japonicu*s first increased and then decreased with increases in the age of female adults. The peak egg volume in the ovary of mated females occurred 2 days earlier than that of virgin females. Within the female age range of 0–15 days, the numbers of eggs at stages I, II, and III first increased and then decreased with increases in the age of female *C. japonicus*, whereas the number of eggs at stage IV increased. The duration of the coexistence of females and males significantly influenced the length and width of the female ovaries, and the longest ovary tube and the highest number of eggs were obtained with a coexistence duration of 0 days. *C. japonicus* female longevity decreased with increases in the number of matings, and the number of eggs laid by females within 15 days decreased with increasing delays in mating. In conclusion, mating can shorten the longevity of *C. japonicus* females, and selecting newly emerged virgin females for mating can significantly improve the number of eggs laid and the breeding efficiency of the parasitoid.

## Introduction

*Parasaissetia nigra* Nietner, one of the species in the main groups of Coccidae^1^, is a notably polyphagous insect that attacks over 80 distinct plant families^2^. This insect is native to Africa, but is currently distributed on six continents except Antarctica^3^. From 2003 to 2008, this pest exploded on a large scale in Yunnan, China, and its occurrence expanded to a cumulative area of 140,000 hm^2^^4^. In recent years, it has emerged in rubber seedlings in Hainan, China. In addition to infecting young leaves of rubber, adults and larvae of this pest can also breed in twigs. The lightly affected rubber trees exhibit fallen leaves, a weaker tree potential, and withered branches, whereas the strongly affected rubber trees experience sooty blotch or even death. Thus, this pest is a threat to forest ecosystems in many areas of the world. The chemical control of *P. nigra* is currently difficult, because it has developed resistance due to the long-term use of pesticides. The abuse of pesticides also pollutes forest ecosystems and destroys biodiversity. Therefore, the biological control using natural enemy, such as parasitoids, are likely the most promising approach to suppress this pest. *Coccophagus japonicus* Compere is a primary, solitary endoparasitoid, its current distribution is in Japan, Nearctic, China and America. *C. japonicus* plays an important role in the biological control of pests belonging to the family Coccidae^5^, particularly *Coccus pseudomagnniliarum* Kuwana, *Ceroplastes floridensis* Comstock, *Ceroplastes japonicus* Green, *Ceroplastes rubens* Maskell, *Saissetia oleae* Olivier, *Coccus hesperidum* L.^6^. The behavior and daily parasitic activity of *C. japonicus* has been detailed by Li et al., including its host distribution, ovipositor penetration, oviposition and postoviposition treatment^7^. Except eggs, 1st instars and adults (black), females of *C. japonicus* can feed on all life stages of *P. nigra* and they exhibit a preference for 3rd instars and adults (brown)^8^. In the laboratory, this endoparasitoid can notably control 3rd instars of *P. nigra*^9,10^.

Reproduction refers to the biological process of producing offspring for the continuation of species, that is, the process of biological production of new individuals^11^. The process of independent development of an animal's fertilized eggs outside the mother's body is called oviparous. Oviparous is characterized by the yolk contained in the egg itself as nutrition during embryonic development. Insects are a kind of oviparous animals, and their embryonic development mainly depends on sufficient yolk protein and other substances accumulated by the developing oocytes, which are used as nutrients necessary for life^12^. Bisexual reproduction is the most basic reproductive method of insects, in the whole reproduction process, mating is an important condition for the reproduction and survival of insect populations^13,14^. The main goal is to fertilize eggs, and this process can enhance genetic diversity. During mating, nutrients are also transferred from males to females with sperm, and these nutrients can be beneficial to the development of females and improve their reproductive performance^15^. However, mating also has some disadvantages to insects. Specifically, much time and energy are needed during mating, and insects are at greater risk of predation during this process^16^. Males are more likely to injure themselves and females during copulation^17–21^. In addition, previous studies have shown that compounds in the seminal fluids of males can reduce female longevity^22,23^. *C. japonicus* is a predominant natural enemy of *P. nigra*, our preliminary study found that mating seriously restricts the oviposition and parasitism of this parasitoid wasp. Therefore, with the aim of controlling *P. nigra*, we examined the effects of *C. japonicus* mating on ovarian development, female longevity and the number of eggs laid by females. These analyses clarify the effects of mating on reproductive performance, provide methods to increase the number of eggs in the parasitic wasp ovary, life span, egg production and parasitism rate, and provide a theoretical basis for the mass reproduction of this parasitoid. So as to lay the foundation for realizing field application of this parasitoid wasp.

## Materials and methods

### Insects

*P. nigra* were collected from rubber trees in proving grounds at the Yunnan Institute of Tropical Crops in 2004 (20° 05′ N, 102° 72′ E). Mass rearing of these insects was performed in the laboratory at 25–27 °C and 70–90% relative humidity, and the insects were fed pumpkin during this process.

*C. japonicus* were collected from the testing ground at the Chinese Academy of Tropical Agricultural Science in Danzhou, Hainan Province (19° 31′ N, 109° 34′ E). After hatching, the insects were reared with *P. nigra* to obtain a sufficiently high population for the experiment. Sex discrimination of *C. japonicus* was performed as described by Askew, Chen and Li^24,25^.

### Experimental procedures and statistical analyses

**Figure 1:**
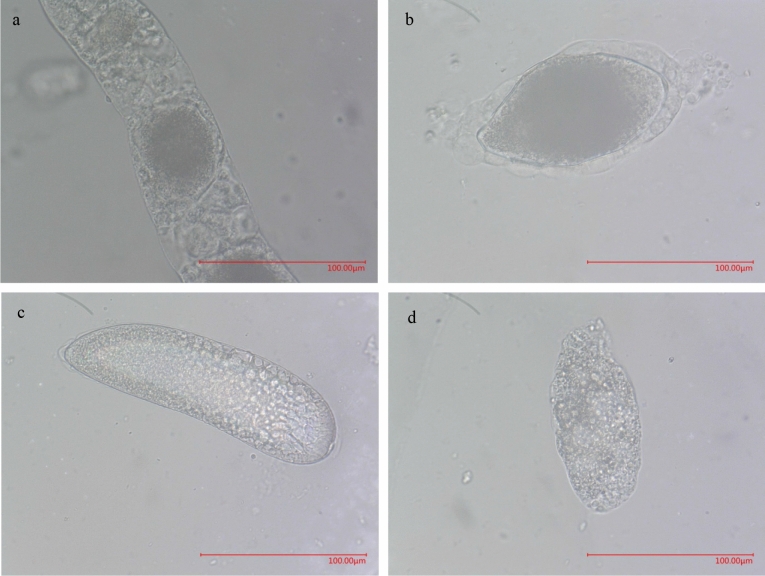
Four egg stages of *C. japonicas.* (**a**) Egg I; (**b**) egg II; (**c**) egg III; (**d**) egg IV.

The following experiments were performed:

1. To determine the effects of mating on the ovarian development of female adults, 10 virgin and 10 mated females were placed inside a clear test tube, which were placed at 27 °C and 70% relative humidity under a 16-h light and 8-h dark photoperiodic regime, and fed a 20% sugar solution on soaked cotton. In total, 20 such test tubes with parasitoid adults were prepared for this experiment. During a 15-day period, one of those 20 test tubes was randomly chosen every 24 h. For each chosen tube, all 10 females were checked, placed in a tube with 75% ethanol (the following experiments were performed under the same above-mentioned environmental conditions), and dissected after their death. An Olympus stereo microscope (SZX16, Japan) was used to observe the reproductive system and egg morphology of female adults of *C. japonicus*. Also, the number of eggs at each stage was recorded. Four stages of C. japonicus eggs were distinguished based on the length of yolk in the egg chamber and the morphological and size features of the eggs (Fig. 1): stage I, early vitellogenic eggs, the yolk is less than 1/2 of the egg chamber length, the eggs have a rounded or elliptical shape, and the egg length is 0.05 mm; stage II, vitellogenic eggs, the yolk is more than 1/2 of the egg chamber length, one end of the egg is larger and the other end is smaller, and the egg length is 0.14 mm; stage III, mature eggs, the yolk is dispersed throughout the egg chamber, the egg length is 0.18 mm, and the egg shape is similar to that of a kidney; stage IV, more mature eggs, yolk thinning is observed, and the egg length is 0.1 mm. Photographs were acquired using an Olympus-SZX16 and Ultra-Depth Three-Dimensional Microscope (VHX-500, Japan). Each treatment was repeated three times.
2. To determine the effects of the coexistence of females and males on the ovarian development of female adults, we placed two females and one male of *C. japonicus* inside a clear test tube. The males were removed from the tube, and the females continued to be fed after the females and males coexisted for 2, 4 and 6 days, respectively. After one week, the females were dissected. We recorded the egg volumes in the ovary and measured the length and width of the ovaries. Females placed in the test tube alone served as the control. All the females were virgins before the experiment, and each treatment was repeated three times.
3. To determine the effects of mating on female longevity and survival, three mating treatments were performed: virgin females: 10 newly emerged virgin females were placed inside a test tube (N = 3); once-mated females: 10 females were placed inside a test tube after one successful mating (N = 3); multiple-mated females (the minimum number of mating times is two): after a successful first mating, the male was replaced, and after a successful second mating, Ten females and ten new males were put into two test tube, five females and five males in each test tube, respectively, new males were replaced every day, until the female died(N = 3). The tubes were checked daily between 8:00 a.m. and 10:00 a.m. local time to count the number of dead females. Each treatment was repeated three times.
4. To determine the effects of delayed mating on the number of eggs laid by females, one newly emerged virgin male was paired with two newly emerged virgin females or with 2-, 4-, and 6-day-old virgin females. Two females and one male of *C. japonicus* insects were placed inside a petri dish. Sixty *P. nigra* (3rd instar) were placed in the dishes for *C. japonicus* parasitization. Every 24 h during a 15-day period, the scales were dissected under a stereomicroscope to determine the number of eggs laid by females and the number of *P. nigra* parasitized, and new scales were regularly replaced every day. Each treatment was repeated three times.
5. To determine the effects of the male age on the number of eggs laid by females, newly emerged virgin females were paired with 1-, 3-, 5- and 7-day-old virgin males. Two females and one male of *C. japonicus* insects were placed into a petri dish. Sixty *P. nigra* (3rd instar) were placed in the dishes for *C. japonicus* parasitization. Every 24 h during a 15-day period, scales were dissected under a stereomicroscope to determine the number of eggs laid by females and the number of *P. nigra* parasitized, and new scales were regularly replaced every day. Each treatment was repeated three times.
6. To determine the effects of mating on the number of eggs laid by female when the host has not been parasitized or has been parasitized, four treatments were performed. A: the part of pumpkin was covered with 30 *P. nigra* (3rd instar) with a transparent plastic cup (the diameter was 7.5 cm, the height was 8.5 cm, the edge of the cup was glued with a circle of sponge, and the bottom of the cup was opened with a 1.1 cm diameter hole), and one mated female was released into the cup; B: one virgin female was released into the cup described above; C: one mated female was released into the cup for 24 h and then removed, 8 days later, another mated female was released into the same cup; D: one mated female was released into the cup for 24 h and then removed, 8 days later, one virgin female was released into the same cup. After 24 h, scales were dissected under a stereomicroscope to determine the number of eggs laid by female. Each treatment was repeated five times.

### Data analysis

The parasitism rate of *C. japonicus* was calculated as the number of parasitized *P. nigra* within the total number of *P. nigra*. Data in figures were stated as the mean ± standard error deviation. Differences between the data were determined by one-way ANOVA and Duncan’s test. Prior to these analyses, all percentage data were transformed using the formula square arcsine transformation. Differences at a probability level of P < 0.05 were considered to be significant in all statistical tests. All the data were analyzed using SPSS 23.0 for Windows (http://www.spss.com). The figures were prepared using Microsoft Excel 2016.

### Terminology

The terminology used in this study followed that of Gaikwad et al. and Boulton et al.^26,27^.

## Results

### Effects of mating on the ovarian development of females

**Figure 2:**
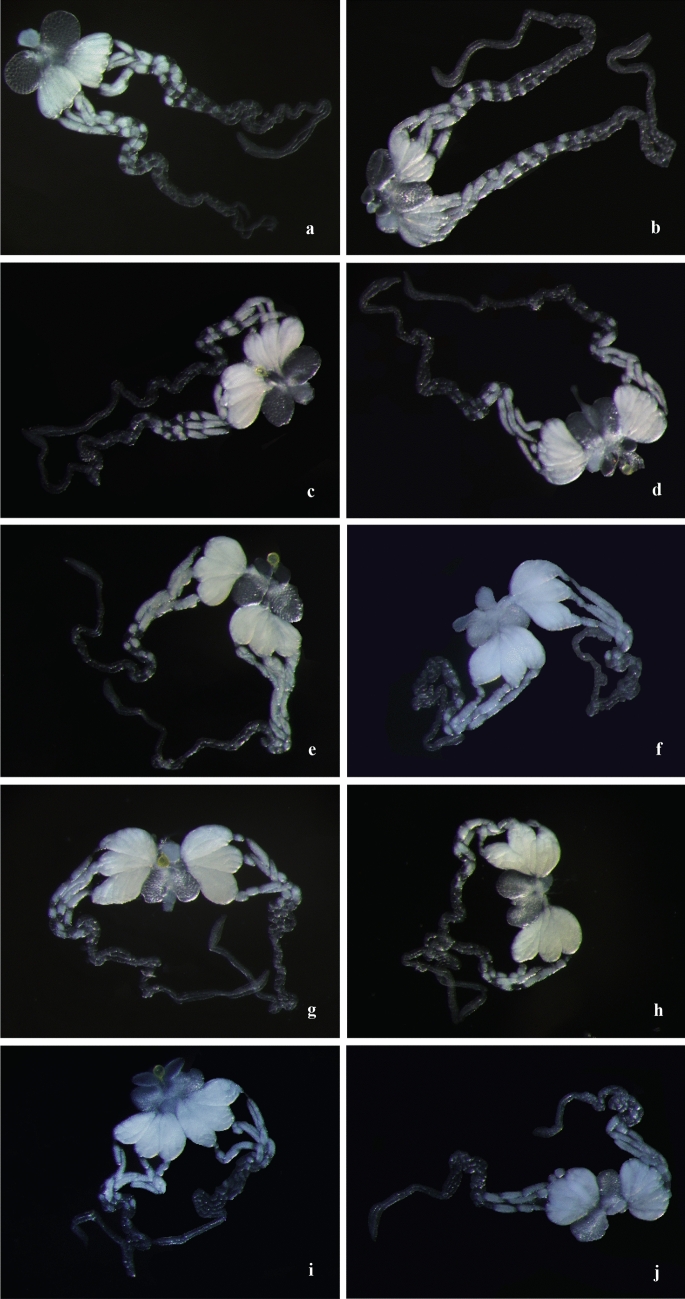
The ovarian development of unmated and mated *C. japonicus* over time after emergence (115 ×). (**a**) The ovary of unmated female that have emerged for 2 h; (**b**) the ovary of mated female that have emerged for 2 h; (**c**) the ovary of unmated female that have emerged e for 1 days; (**d**) the ovary of mated female that have emerged for 1 days; (**e**) the ovary of unmated female that have emerged for 5 days; (**f**) the ovary of mated female that have emerged for 5 days; (**g**) the ovary of unmated female that have emerged for 10 days; (**h**) the ovary of mated female that have emerged for 10 days; (**i**) the ovary of unmated female that have emerged for 15 days; (**j**) the ovary of mated female that have emerged for 15 days.

Two hours after emergence, the eggs moved to the ovary, and these eggs mainly consisted of stage-I and stage-II eggs (Fig. 2a,b). In 1-day-old females, the numbers of eggs at stage I and II near the ovary decreased in number, and the number of eggs at stage III increased (Fig. 2c,d). The ovaries were fuller in 1- to 4-day-old females (Fig. 2). After 4 days of age, the size of the ovary decreased, and the number of eggs in the ovariole increased with increasing female age. No significant difference in ovarian development was found between mated and virgin females.

**Figure 3:**
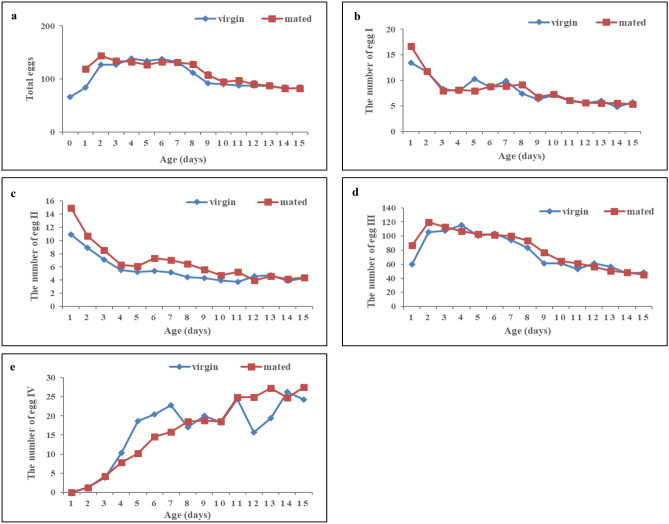
The number of *C. japonicus* eggs at four egg stages of at different ages. Age: the number of days after the emergence of females.

The peak egg load in the ovary of mated females occurred 2 days earlier than that of virgin females. The peak egg load in the ovaries of mated and virgin females were observed 2 and 4 days after emergence, respectively, and the egg load in the ovaries were 143.6 and 139.0 eggs, respectively. After 4 days of age, the egg load in the ovary decreased with increasing age. The egg load in the ovary of virgin females decreased with increases in age from 7 to 9 days, whereas that of mated females decreased rapidly from 8 to 10 days of age (Fig. 3a).

The numbers of eggs at stages I and II were highest in virgin or mated females aged 1 day. At this age, the numbers of eggs at stage I in mated and virgin females were 16.7 and 13.5, respectively, and those at stage II were 14.9 and 10.9, respectively. At later ages, the number of eggs at stages I and II decreased with increases in the female age. In 15-day-old mated and virgin females, the numbers of eggs at stage I were 5.4 and 5.7, respectively, and those at stage II were 4.4 and 4.3, respectively (Fig. 3b,c).

The number of eggs at stage III in virgin or mated females first increased and then decreased with increasing age. The highest number of eggs at stage III in mated and virgin females was obtained at ages of 2 days (119.8) and 44 days (115.3), respectively. After these ages, the number of eggs at stage III decreased with increasing female age. The lowest number of eggs at stage III was found in 15-day-old mated and virgin females (45.4 and 48.5, respectively) (Fig. 3d).

A positive relationship was found between the number of eggs at stage IV and the female age. No eggs at stage IV were found in 1-day-old mated or virgin females, and the highest number of eggs at stage IV was found in 15-day-old mated females (27.5) and 14-day-old virgin females (26.2) (Fig. 3e).

### Effects of coexistence of females and males on ovarian developments

**Figure 4:**
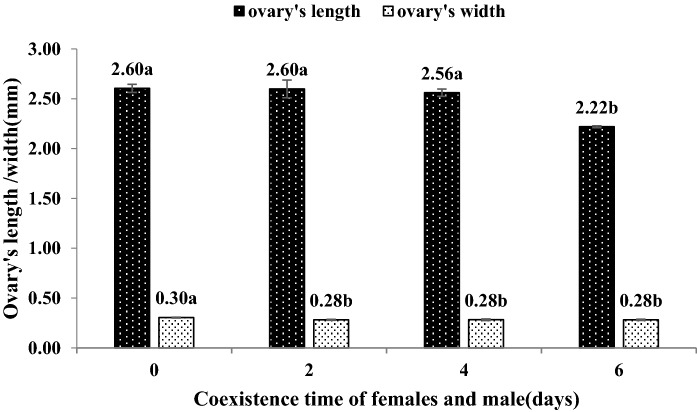
The length and width of *C. japonicus* ovary at different coexisting time of female and male adults. Data in the figure is mean ± standard error, different letters indicate significant differences in Duncan’s test, with a probability of 5%.

The time of coexistence of females and males significantly influenced the length and width of the female ovaries (F = 12.59, P < 0.05; F = 3.16, P < 0.05). Specifically, ovary lengths of 2.60, 2.60 and 2.56 mm were obtained after the females and males coexisted for 0, 2 and 4 days, respectively, and these three lengths were not significantly different. An ovary length of 2.22 mm was obtained after the females and males coexisted for 6 days, and this length was significantly longer than that obtained with the coexistence time. The width of the ovary was 0.33 mm when the females and males coexisted for 0 days, and this width was significantly larger than those obtained with a coexistence time of 2, 4 and 6 days (the width obtained with these treatments was 0.28 mm) (Fig. 4).

**Figure 5:**
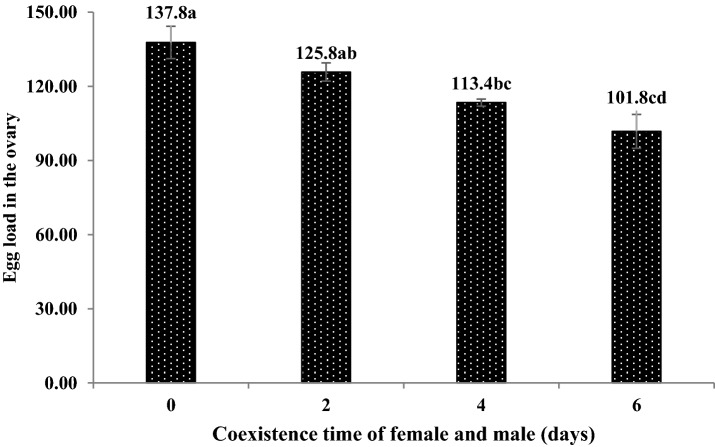
The egg load in the ovary of *C. japonicus* at different coexisting time of female and male adults. Data in the figure is mean ± standard error, different letters indicate significant differences in Duncan’s test, with a probability of 5%.

The female’s egg load in the ovary significantly decreased with increases in the coexistence time (F = 9.15, P < 0.05). Specifically, an egg load in the ovary of 137.8 was obtained if the females and males coexisted for 0 days, whereas egg load in the ovary of 125.8, 113.4 and 101.8 were obtained with coexistence time of 2, 4 and 6 days, respectively. The egg volumes in the ovary obtained with a coexistence time of 0 d was significantly higher than those obtained with a coexistence time of 4 and 6 days (Fig. 5).

### Effects of mating on female longevity and survival

**Figure 6:**
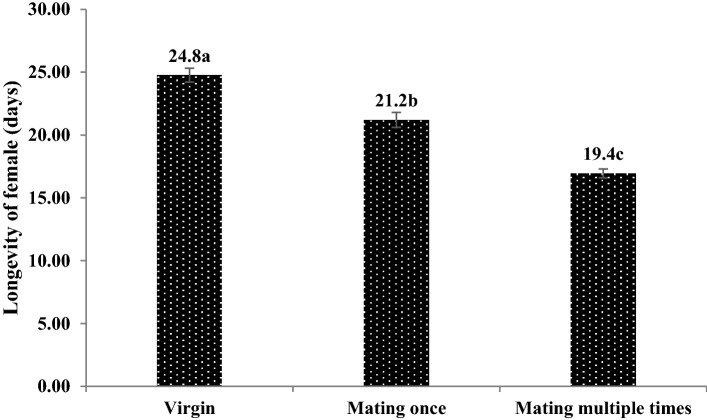
The longevity of female *C. japonicus* at different mating times. Data in the figure is mean ± standard error, different letters indicate significant differences in Duncan’s test, with a probability of 5%.

**Figure 7:**
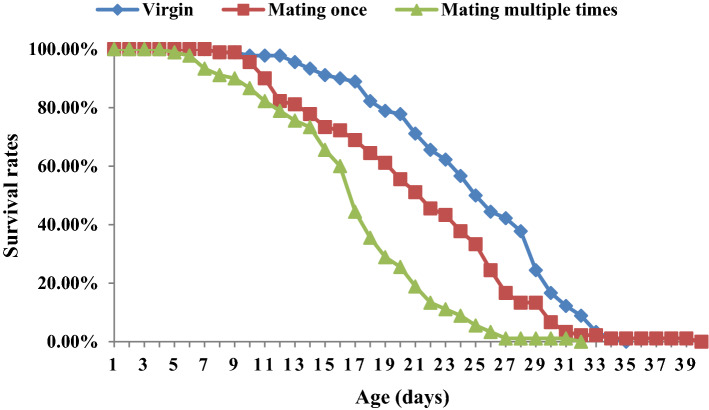
The survival rates of female *C. japonicus* at different mating times. Data in the figure is mean ± standard error, different letters indicate significant differences in Duncan’s test, with a probability of 5%.

The female longevity and survival decreased with increases in the number of times that *C. japonicus* females mated. The female longevity of virgin, once-mated and multiple-mated females were 26.0, 21.7 and 16.5 days, respectively, and the differences between these groups were significant (F = 80.98, P < 0.001) (Figs. 6 and 7).

### Effect of delayed mating on the number of eggs laid by females

**Figure 8:**
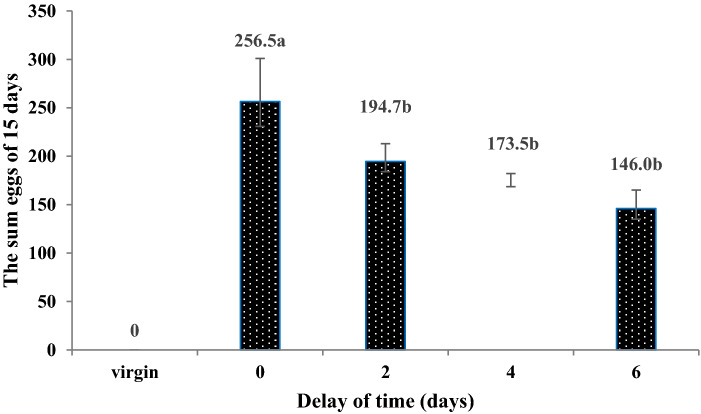
The number of eggs laid by females *C. japonicus* at different delayed mating. Data in the figure is mean ± standard error, different letters indicate significant differences in Duncan’s test, with a probability of 5%.

**Figure 9:**
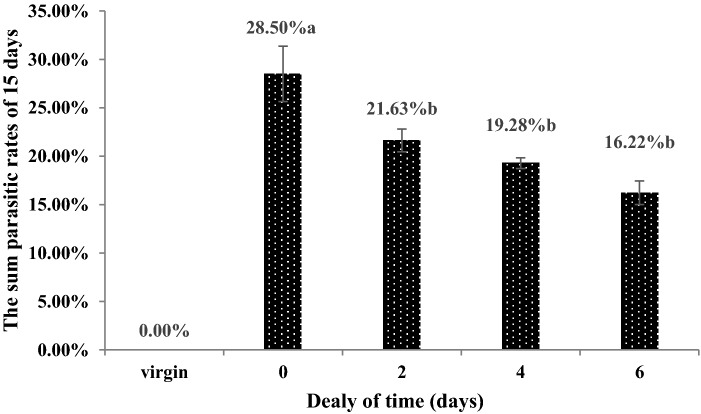
The parasitic rates of females *C. japonicus* at different delayed mating. Data in the figure is mean ± standard error, different letters indicate significant differences in Duncan’s test, with a probability of 5%.

The number of eggs laid by females and parasitism rates within 15 days decreased with increasing delays in mating. More eggs were laid and higher parasitism rates with no mating delay (257 eggs and 28.50%) than with any other treatment, i.e., treatments in which the mating was delayed by 2 (195 eggs and 21.63%), 4 (174 eggs and 19.28%), and 6 days (146 eggs and 16.22). Females did not lay eggs when they had not undergone mating. However, when the mating was delayed by 2, 4 and 6 days, the number of eggs laid by the females and parasitism rates was not significantly different (Figs. 8 and 9).

### Effects of male age on the number of eggs laid by females

**Figure 10:**
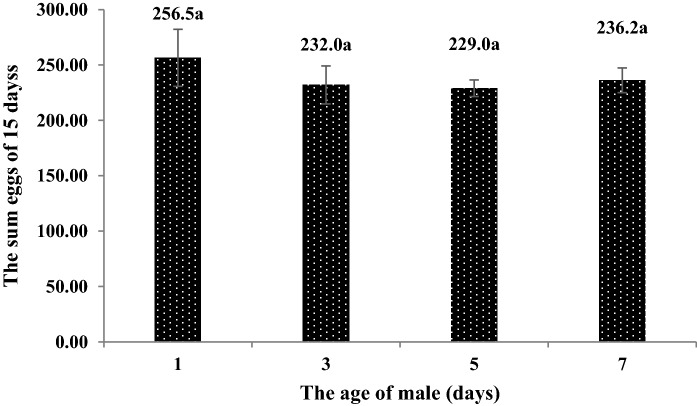
The number of eggs laid by females *C. japonicus* at different male ages. Data in the figure are mean ± standard error, different letters indicate significant differences in Duncan’s test, with a probability of 5%.

**Figure 11:**
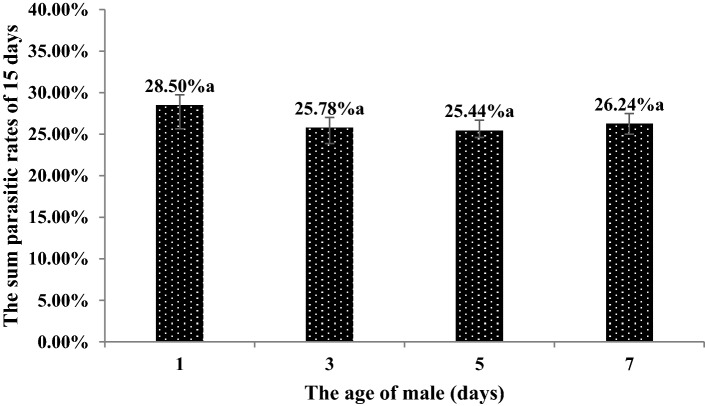
The parasitic rates of females *C. japonicus* at different male ages. Data in the figure are mean ± standard error, different letters indicate significant differences in Duncan’s test, with a probability of 5%.

The highest number of eggs laid by females and parasitism rates within 15 days were obtained by mating with 1-day-old males. However, no significant differences were found between the treatments (F = 0.543, P > 0.05) (Figs. 10 and 11).

### Effect of mating and hosts on the number of eggs laid by females

**Figure 12:**
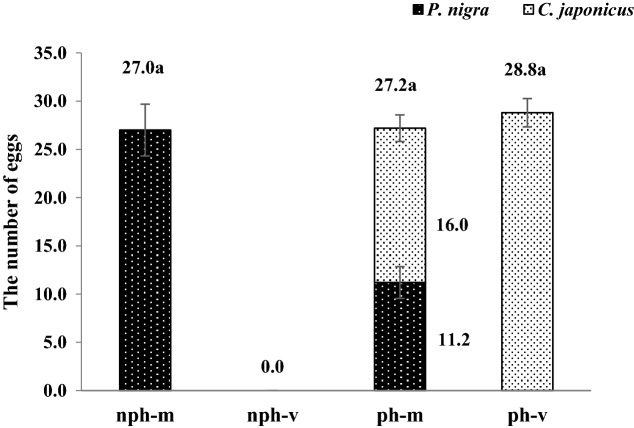
The number of eggs laid by virgin and mated female *C. japonicus* at different hosts. Data in the figure are mean ± standard error, different letters indicate significant differences in Duncan’s test, with a probability of 5%; nph-m: when the host has not been parasitized, the number of eggs laid by a mated female; nph-v: when the host has not been parasitized, the number of eggs laid by a virgin female; ph-m: when the host has been parasitized, the number of eggs laid by a mated female; ph-v: when the host has been parasitized, the number of eggs laid by a virgin female; *P. nigar*: the number of eggs laid in *P. nigar* by a female; *C. japonicus*: the number of eggs laid in *C. japonicus* by a female.

Mated females could lay eggs in *P. nigra* that had not been parasitized, *P. nigra* that had been parasitized, and the larvae of *C. japonicus* parasitized in the *P. nigra*, the spawning volume was 27.0, 11.2, and 16.0 respectively. Virgin females could not lay eggs in *P. nigra* that had not been parasitized and *P. nigra* that had been parasitized, but could lay eggs in the larvae of *C. japonicus* parasitized in the *P. nigra*, the number of eggs laid was 28.8.There was no significant difference in the total number of eggs laid by the females between different treatment (Fig. 12).

## Discussion

Based on the different mechanisms of egg maturation after the hatching of females, Flanders distinguished two types of parasitoid wasps: synovigenic and proovigenic parasitoids^28^. The term “synovigenic” means that the ovary of newly emerged virgin females contains no or only a few mature eggs, and thus, females need to acquire nutrients to continue the development of their eggs. In contrast, the term “proovigenic” means that the eggs in the ovaries of newly emerged virgin females have completely or almost completely matured, and thus, females usually do not need to acquire nutrients. A few mature eggs were found in the ovaries of newly emerged virgin *C. japonicus* females, and the number of eggs increased with increases in the female age, which indicates that the parasitoid is synovigenic. Previous studies have classified eggs into three stages according to the length of the yolk in the egg chamber and the morphological and size features of the eggs^26,29,30^. In this study, we distinguished four stages of *C. japonicus* eggs. The morphological features of eggs at stages I, II, and III are similar to those of *Pteromalus puparum*, whereas the yolk of eggs at stage IV was thinning and had a relatively small volume.

Some species of parasitoid wasps use lipids and proteins stored in the larval stage for egg development when only providing carbohydrates. In the absence of host parasitism, parasitoid wasps gain energy by resorbing eggs to search for hosts, survive and lay eggs^31–33^. In this study, within the female age range of 0–15 days, increases in the female *C. japonicus* age were associated with reductions in the number of mature eggs (stage III), increases in the number of eggs at stage IV and reductions in the egg volume in the ovary, which indicated that egg resorption might have occurred in *C. japonicus*. In different parasitoid wasp species, when mature eggs are reabsorbed, faded and broken shells can sometimes be clearly observed^34^. We did not find any eggshell left by resorbed eggs in the ovary of *C. japonicus*, and this phenomenon also occurs in Encyrtidae^35^. In this study, the peak egg volume in the ovaries of mated females occurred 2 days earlier than that of virgin females. This result indicates that mating can promote egg development but has no obvious effect on the total number of eggs. Klowden and Chambers proposed that males may import a substance into females during mating, and this substance may be used to promote egg development by regulating a certain substance that already exists in females, thereby making eggs generated^36^. Whether the promotion of the ovarian development of Japanese scale aphid wasps in mating is due to a coordinated substance in drone semen remains to be further studied.

The number of eggs laid after multiple matings exhibits significant differences among different insects. Multiple mating increases fecundity in most insects, such as *Coelopa frigida*, *Papilio xuthus*, and *Chrysochus cobaltinus*^37–40^, whereas in some insects, such as *Utetheisa ornatrix* and *Nysius huttoni*, multiple matings have no effect on fecundity^41,42^. In addition, in *Plutella xylostella* Linnaeus, *Histiostoma feroniarum* Dufour, among others, the number of eggs laid decreased with increases in the number of matings^43–45^. In many species, mating directly affects female longevity. For example, in *Eretmocerus hayati* Girault & Dodd and *Encarsia sophia* Girault & Dodd, the longevity of mated females is significantly lower than that of virgin females, and the female longevity decreases with increasing number of matings^46^. This phenomenon also occurs in *C. japonicus*. Mating leads to shorter insect longevity for several reasons, including reduced nutrient intake, bacterial infection and consumption of energy during mating^16,18,19,21,47^. However, in some insects, such as *Monochamus alternatus* Hope, mating has no effect on longevity^48^.

Delayed mating means that insects can’t mate at normal time due to a variety of environmental factors^49^, and this behavior is very common in insects. Delayed mating has a significant influence on the mating rate, preoviposition time, oviposition time, number of eggs laid, and egg viability in many insects, such as *Epiphyas postvittana* Tortricidae, *Plodia interpunctella* Hübener, *Mnesampela private* Guenee, and *Nauphoeta cinerea* Olivier, in which delayed mating reduces fecundity^50–55^. In this study, delayed mating of *C. japonicus* reduced the number of eggs laid and increased the preoviposition time. However, in some insects, such as *Eurema hecabe* L. and *Dasylepida ishigakiensis* Niijima & Kinoshita, delaying the mating of females as opposed to that of males does not have a notable impact on the number of eggs laid^56,57^, and as the number of mating increases, the longevity decreases. Unmated femalesdid not able to lay eggs in an unparasitised host.

Previous study found some parasitoid wasps that develop heterogeneously, such as *Encarsia japonica* Viggiani^58^. The bisexually reproduced eggs are oviposited in the non-parasitized host and develop into female offspring, whereas the parthenogenetic eggs are oviposited in the same or heterogeneous primordial parasitoid larvae in the host and develop into male offspring. In our study, mated females of *C. japonicus* could lay eggs in unparasitized and parasitized *P. nigra*, as well as *C. japonicus* larvae in *P. nigra*. Virgin females could lay eggs in neither unparasitized nor parasitized *P. nigra*, but could lay eggs in the larvae of *C. japonicus* parasitized in the *P. nigra*. However, the specific gender of the offspring still needs to be further studied.

In conclusion, mated *C. japonicus* females had a significantly reduced longevity than unmated females, and as the number of mating increases, the longevity decreases. Unmated females were not able to lay eggs in a non-parasitized host, but they could lay eggs in the larvae of *C. japonicus* larvae in *P. nigra*. The number of eggs laid was significantly reduced by delaying the mating of female *C. japonicus*, whereas the age of the males within the range of 0–7 days had no significant effect on the total number of eggs laid by newly emerged females, which indicates that there were no significant differences in mating ability and quality among 0–7-day-old *C. japonicus* males. Thus, breeding *C. japonicus* in the laboratory, for biological control of *P. nigra* can be improved according to current knowledge. Newly emerged virgin females and 0–7-day-old males should be used for mating, and the mating time should be control as less as possible to increase the mating rate, parasitism rates and female longevity, so as to better improve the indoor breeding efficiency.
